# Toward ICE-XRF fusion: real-time pose estimation of the intracardiac echo probe in 2D X-ray using deep learning

**DOI:** 10.1007/s11548-025-03493-z

**Published:** 2025-08-18

**Authors:** Annelies Severens, Midas Meijs, Vipul Pai Raikar, Richard Lopata

**Affiliations:** 1 Biomedical Engineering, University of Technology Eindhoven, Groene Loper 3, 5612 AE Eindhoven, The Netherlands; 2 IGT Systems, Philips Healthcare, Veenpluis 6, 5684 PC Best, The Netherlands; 3 IGT Devices, Philips Healthcare, 222 Jacobs St., Cambridge, MA 02138 United States of America

**Keywords:** Valve repair, Intracardiac echocardiography, Image-guided therapy, Medical deep learning, Object detection, Pose estimation

## Abstract

****Purpose**:**

Valvular heart disease affects 2.5% of the general population and 10% of people aged over 75, with many patients untreated due to high surgical risks. Transcatheter valve therapies offer a safer, less invasive alternative but rely on ultrasound and X-ray image guidance. The current ultrasound technique for valve interventions, transesophageal echocardiography (TEE), requires general anesthesia and has poor visibility of the right side of the heart. Intracardiac echocardiography (ICE) provides improved 3D imaging without the need for general anesthesia but faces challenges in adoption due to device handling and operator training.

****Methods**:**

To facilitate the use of ICE in the clinic, the fusion of ultrasound and X-ray is proposed. This study introduces a two-stage detection algorithm using deep learning to support ICE-XRF fusion. Initially, the ICE probe is coarsely detected using an object detection network. This is followed by 5-degree-of-freedom (DoF) pose estimation of the ICE probe using a regression network.

****Results**:**

Model validation using synthetic data and seven clinical cases showed that the framework provides accurate probe detection and 5-DoF pose estimation. For the object detection, an F1 score of 1.00 was achieved on synthetic data and high precision (0.97) and recall (0.83) for clinical cases. For the 5-DoF pose estimation, median position errors were found under 0.5mm and median rotation errors below $7.2^{\circ }$.

****Conclusion**:**

This real-time detection method supports image fusion of ICE and XRF during clinical procedures and facilitates the use of ICE in valve therapy.

## Introduction

Valvular heart disease impacts millions of people worldwide, affecting 2.5% of the general population and 10% of the individuals aged over 75 [1, 2]. Despite the high prevalence of valve disease and its association with increased mortality, the high risks of traditional surgery leave many patients untreated [3].

Transcatheter therapies provide a less invasive and effective alternative to surgery for valve repair [4]. This has opened up access to treatment for patients who did not meet the inclusion criteria for surgical interventions. The success of these procedures relies on high-quality imaging throughout the procedure for accurate device placement and procedural guidance [5] . X-ray fluoroscopy (XRF) is commonly used to visualize surgical instruments, while ultrasound imaging provides anatomical context. In current clinical practice, transesophageal echocardiography (TEE) is the gold standard ultrasound modality for valve interventions. However, its use is limited by the intolerance of certain patients to general anesthesia and the challenges in imaging the right side of the heart due to the position of the tricuspid valve and interference, such as acoustic shadowing, of previously placed prosthetic implants [6].

Real-time three-dimensional (3D) intracardiac echocardiography (ICE) has recently emerged as a powerful tool in valve procedures [7–9]. ICE utilizes a small form factor ultrasound transducer with a transducer window of 3 mm by 2 cm that allows the user to image the heart from within, offering high-resolution real-time visualization of the valve leaflets. Unlike TEE, ICE provides superior visualization of the complex 3D anatomy, which is crucial to guide catheter-based interventions [10]. In addition, ICE reduces the need for general anesthesia, as the probe can be inserted through the femoral artery or vein, making it suitable for a wider range of patients [11].

Although ICE offers imaging advantages, its adoption in clinical practice has been slow due to factors such as required operator training and complex device manipulations. A potential solution to facilitate the adoption of ICE is the fusion of ICE and XRF images, providing a comprehensive view of cardiac anatomy and surgical tools in a single display. The integration simplifies navigational tasks and procedures, potentially allowing single-operator interventions [12]. This approach may reduce procedural times and make the use of ICE in valve interventions more accessible.

Image fusion combines imaging data from multiple modalities to provide comprehensive diagnostic insights [13]. Valve repair procedures benefit from this approach, as it improves navigation by aligning real-time ultrasound with X-ray imaging for better procedural guidance. For effective image fusion, precise localization and real-time tracking of the ultrasound probe within the X-ray coordinate system are required. Tracking algorithms to accurately detect and continuously monitor the probe’s position and orientation are essential in this process for ideal head-eye coordination.

### Related work

Similar imaging challenges have been addressed in cardiac procedures, such as mitral valve repair, where fusion of TEE and XRF has become standard practice to enhance anatomical visualization. Fusion is typically achieved through pose estimation and registration techniques. Various methods have been investigated and developed for TEE fusion, such as electromagnetic tracking systems (EM) [14], marker-based registration [15], and image-based algorithms [16, 17]. EM tracking and marker-based approaches require additional hardware, complicating their integration into the clinical workflow. In contrast, image-based algorithms, especially those that use deep learning, have shown promise for marker-free real-time fusion by improving both accuracy and speed [18, 19].

Related work for tracking of the ICE probe is limited. The integration of ICE with XRF presents additional challenges as a result of the increased flexibility and variability of the ICE probe. An early attempt was made by Ralovich et al. [20], where six degrees of freedom (DoF) were estimated using fiducial markers. This demonstrated a proof-of-concept but required additional hardware, lacked real-time capabilities, and relied on traditional machine learning. More recently, Annabestani et al. [21] developed a deep learning framework to predict the roll angle of the ICE probe using biplane X-ray imaging. However, the use of biplane imaging remains uncommon in transcatheter valve procedures, limiting the generalizability of the proposed solution. Moreover, the focus on a single DoF leaves room for further development to estimate the full pose of the ICE probe.

### Aim of study

This paper proposes a two-step deep learning framework for real-time estimation of the 5-DoF pose of the ICE probe in 2D XRF images. First, the probe is coarsely detected using an object detection network, followed by a refinement step to estimate its pose. This method supports ICE-XRF image fusion in clinical procedures, enhancing procedural accuracy, reducing operator dependency, and expanding the use of minimally invasive valve repair treatments.

## Materials and methods

### Synthetic data generation

**Fig. 1:**
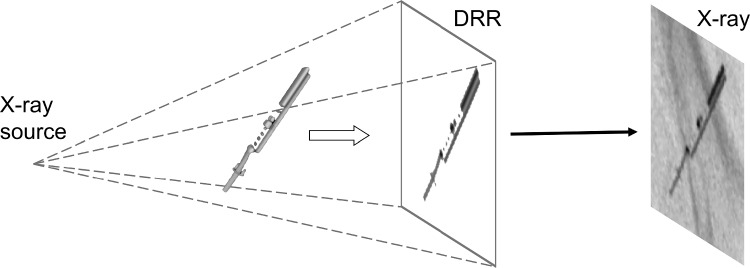
Schematic overview of the Digital Reconstructed Radiograph (DRR) framework

Due to the limited availability of labeled X-ray data for ICE probe procedures, synthetic datasets were created using digital reconstructed radiographs (DRRs). DRRs were generated by simulating X-ray projections from 3D CBCT models, acquired with rotational angiography, of the ICE probe in various orientations, similar to Heiman et al. [22]. These projections were combined with clinical X-ray images featuring common endovascular devices, such as wires and catheter sheaths. The X-ray images were collected from edge-to-edge repair procedures, both mitral and tricuspid, aortic valve repair procedures, and left atrial appendage closures, from 21 medical centers and 118 patients. All procedures were transcatheter interventions. The ICE projections were merged by masking the probe head and resampling to the X-ray image resolution. Smoothing was applied to the projections to ensure smooth transition of the edges. The DRR generation process is schematically shown in Fig. 1.

Two separate datasets were created for the detection and pose estimation tasks. Each dataset contained 8,000 training images, 2,000 validation images, and 1,000 test images. The detection dataset contained 512x512 images with pixel sizes ranging from 0.25mm to 0.55mm, while the pose estimation dataset included 100x100 pixel patches cropped around the ICE probe, with similar pixel size variations.

### Clinical data

In total, seven clinical cases were obtained from valve procedures performed in several hospitals in the USA, using different C-arm systems, among which are Philips Allura and Azurion X-ray systems (Philips Healthcare, NL). The 3D ICE probe that was focused on was the VeriSight Pro (Philips Healthcare, NL). In the X-ray images, various clinical objects were present, including the TEE probe, wires, catheter sheaths, and other surgical instruments. These cases were used for clinical validation of the proposed framework, mentioned in Sect. 2.7.

### Model overview

**Fig. 2:**
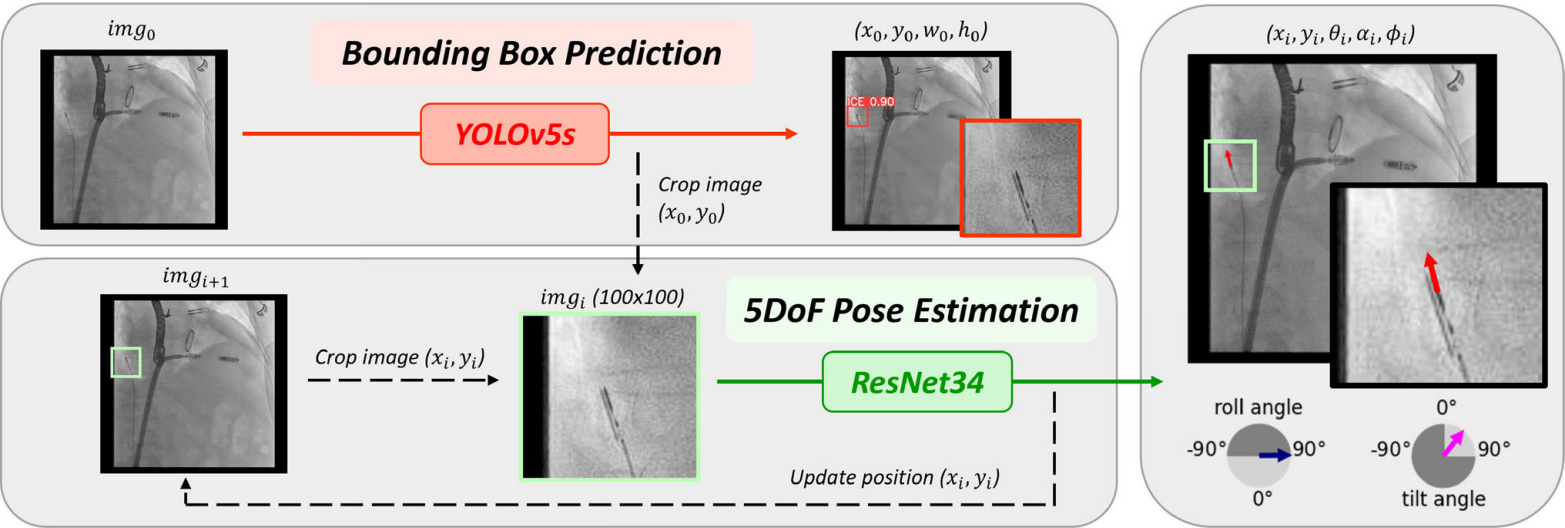
Overview of the two-step framework: (upper) detection module, where bounding box prediction is performed to localize the ICE probe, and (lower) pose estimation module, where the predicted midpoint of the first module is used to crop the original input image ($img_i$) and the 5-DoF pose is estimated

The proposed two-step framework is outlined in Fig. 2. First, a coarse detection of the ICE probe is performed by predicting its bounding box, as described in Sect. 2.5. This step focuses on the ICE probe, minimizing background disturbances from surgical equipment and other foreign objects similar to the probe.

In the next step, the midpoint of the bounding box ($x_0,y_0$) is used to crop the image ($img_0$) to a patch of 100x100 pixels. This reduces noise and lowers computational costs for the 5-DoF pose estimation, which is described in Sect. 2.6.

### Pose parameters

**Fig. 3:**
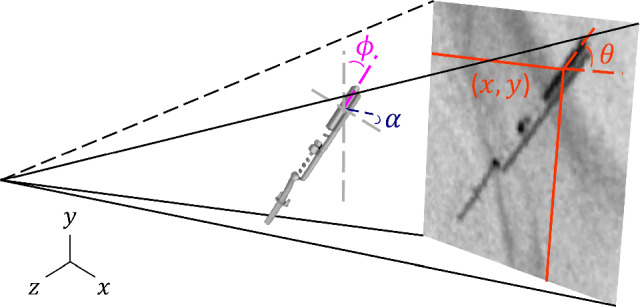
Definition of the 5-DoF pose parameters describing the orientation of the ICE probe

The estimated pose parameters consist of in-plane and out-of-plane parameters, which are shown in Fig. 3.

**Fig. 4:**
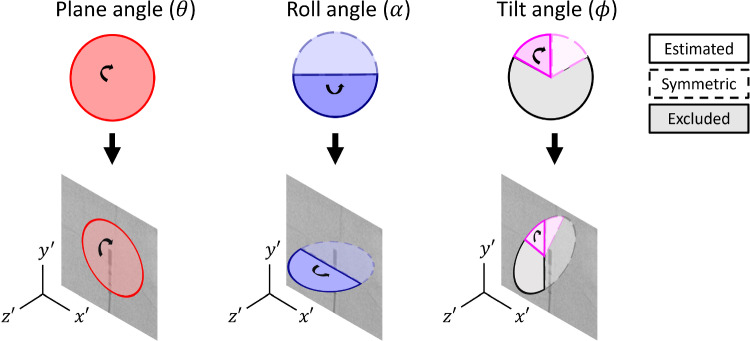
Overview of the definitions and restrictions of the rotational parameters of the ICE probe defined in the 2D X-ray image coordinate system

The in-plane parameters include the positional coordinates of the center of the transducer window (*x*, *y*) and the plane angle ($\theta$), which represents the rotation within the 2D X-ray plane and can be fully recovered from 2D X-ray data (Fig. 4).

The out-of-plane parameters include the remaining two rotational parameters: the roll angle ($\alpha$), describing rotation around the probe’s axis, and the tilt angle ($\phi$), representing inward/outward tilt relative to the X-ray projection (Fig. 4). Due to the symmetry of the probe, only half of the rotation range for both roll and tilt angles can be recovered from the 2D X-ray data. Tilt angles larger than $\pm$ $60^{\circ }$ cause significant foreshortening and are unlikely in clinical settings. Therefore, the tilt angles were constrained within this range for relevance.

### Detection model

Detection of the ICE probe using object detection was performed as an initial step in the framework. This step focuses on localizing the ICE probe to minimize background disturbances and facilitate the subsequent pose estimation network.

The YOLOv5 object detection network, pretrained on the COCO dataset [23], was used to predict bounding boxes around the probe head [24]. Among the YOLOv5 variants, the *small* model was selected, using one-third of the original depth and half of the number of layers compared to the original.

To improve the generalization, a range of data augmentation techniques was applied. These included random brightness and contrast adjustments, translations (±10%), scaling (±50%), and horizontal flipping to introduce spatial diversity. Additionally, Gaussian and Poisson noise models were added to mimic X-ray artifacts. Gaussian noise was applied with a standard deviation ranging from 0 to 1. Poisson noise was applied by first scaling the image intensity between 0.8 and 1.2, which was used to sample from the Poisson distribution. The image was rescaled using the original scale factor to maintain the original intensity range. Augmentations were applied with a 30% probability, and all images were normalized to maintain consistency across the dataset.

The model was trained on images of 512x512 pixels with a batch size of 32 for 300 epochs with an early stopping criterion of no improved validation accuracy for 100 epochs. The complete intersection of union (CIoU) was used as loss optimization for bounding box regression [25], while the binary cross-entropy (BCE) was used for both classification and objectness scores [26]. Training used a stochastic gradient descent (SGD) optimizer [27], an initial learning rate of $10^{-2}$ and a cosine annealing scheduler for gradual learning rate reduction [28].

### Pose estimation model

Pose estimation was performed to find the full orientation of the ICE probe. A localized patch cropped using the output of the detection model (Sect. 2.5) is used as input to predict the 5-DoF pose of the ICE probe.

The model is based on a ResNet34 architecture, pretrained on ImageNet [29], which was modified for regression by removing its final fully connected layer and adapting its output to represent the five pose parameters: $x, y, \theta , \alpha , \phi$, as described in Sect. 2.4. Position parameters were normalized for scale-invariant predictions, while rotational parameters were reformulated for efficient convergence. The plane angle was represented as a two-dimensional vector with decomposed *x*- and *y*-components. The roll angle was parameterized as a relative Euler angle in the range [$-90^{\circ }$, $90^{\circ }$] and normalized between 0 and 1. The tilt angle was captured by the cosine of the direction vector, ranging from 0.5 to 1.0, representing the restricted tilt range of $60^{\circ }$ and $0^{\circ }$, respectively.

Augmentations similar to Sect. 2.5 were applied with 20% probability, and all images were normalized for consistency.

To model used $L_1$ loss with weighted emphasis on the rotational parameters for better accuracy to account for their increased prediction complexity, see Equation 1. Loss weights were set to [$w_1$=1, $w_2$=2, $w_3$=6, $w_4$=3], for the position, the plane angle, the roll angle, and the tilt angle, respectively. These values were selected through a heuristic approach, where different combinations were tested on a validation set, and the configuration yielding the best overall pose estimation performance was chosen. The training was carried out with a batch size of 32 for 300 epochs using model checkpointing, where after training the checkpoint with the lowest validation loss is selected. The Adam optimizer was used and initialized with a learning rate of $10^{-4}$ [30].

1 $$\begin{aligned} L_{total} = w_1 L_{x,y} + w_2 L_{\theta } + w_3 L_{\alpha } + w_4 L_{\phi } \end{aligned}$$

### Clinical validation

The models were validated on clinical images from seven clinical cases, described in Sect. 2.2. The clinical validation set contained 1,700 randomly selected and manually annotated images. The images were annotated in three steps: (1) annotating the position by marking the midpoint of the transducer window, (2) annotating the plane angle using two points along the probe head, and (3) annotating the roll and tilt angles by visually matching a 3D template of the device.

### Evaluation metrics

Annotation robustness was assessed using inter- and intra-user variability on 20 synthetic samples. Three users annotated the dataset for inter-user variability, while one user annotated three times at different points in time for intra-user assessment. Accuracy was measured by comparing mean intra-user annotations to ground-truth values.

Object detection was evaluated using precision, recall, and F1 score. True and false positives were determined using an intersection over union (IoU) threshold of 0.4, as the detection model is not focused on the exact location.

The performance of the pose estimation model was evaluated using the absolute error in all pose parameters, as the direction of the errors is not considered significant in this study. As a normal distribution cannot be assumed, the median was reported, along with the $10^{th}$ and $90^{th}$ percentiles to assess the accuracy of the model. To assess the model’s robustness across different image resolutions, statistical significance was evaluated using p-values between test sets separated into three pixel size groups, ${px}_{size}$ $<$0.3mm, 0.3mm<$px_{size}$<0.5mm, and $px_{size}$>0.5mm.

## Results

### User variability

The intra- and inter-user variability of the annotations of the five pose parameters is shown in Table 1. Both inter- and intra-user variability reveals comparable values, indicating consistent and aligned performance across users.

**Table 1:** Absolute errors of inter- and intra-user variability and user error of the annotations

	x (mm)	y (mm)	$\theta$ ($^{\circ }$)	$\alpha$ ($^{\circ }$)	$\phi$ ($^{\circ }$)
Intra-user variability	0.1 [0.0, 0.1]	0.1 [0.0, 0.1]	0.6 [0.2, 2.3]	6.7 [1.1, 12.4]	4.5 [0.4, 22.7]
Inter-user variability	0.1 [0.0, 0.2]	0.1 [0.0, 0.2]	0.8 [0.2, 2.8]	6.5 [1.7, 18.1]	5.4 [1.5, 17.37]
User error	0.2 [0.1, 0.3]	0.2 [0.1, 0.4]	1.9 [0.2, 3.5]	4.9 [0.6, 15.0]	12.2 [2.5, 22.3]

**Table 2:** Absolute errors for the 5-DoF pose estimation: prediction errors on synthetic and clinical test data with median and [$10^{\text {th}}$, $90^{\text {th}}$] percentile values, separated by pixel size

Test data	Pixel size (mm)	x (mm)	y (mm)	$\theta$ ($^{\circ }$)	$\alpha$ ($^{\circ }$)	$\phi$ ($^{\circ }$)
	${px}_{size}<0.3$	0.2 [0.0, 0.6]	0.3 [0.0, 0.6]	1.0 [0.2, 2.9]	3.6 [0.8, 12.3]	4.7 [0.8, 19.5]
	0.3$<$ ${px}_{size}<0.5$	0.4 [0.1, 1.4]	0.3 [0.1, 1.4]	1.3 [0.2, 6.7]	5.3 [1.1, 26.8]	4.4 [0.9, 14.9]
	${px}_{size}>0.5$	0.7 [0.1, 3.1]	0.6 [0.1, 5.2]	1.6 [0.2, 15.6]	7.5 [1.4, 69.0]	4.8 [1.3, 21.3]
Synthetic	all	0.4 [0.1, 1.3]	0.3 [0.1, 1.3]	1.2 [0.2, 6.3]	5.3 [1.1, 24.3]	5.3 [0.9, 18.1]
	${px}_{size}<0.3$	0.3 [0.1, 0.8]	0.4 [0.1, 1.2]	1.5 [0.3, 5.1]	9.6 [1.6, 35.1]	*
	0.3$<$ ${px}_{size}<0.5$	0.3 [0.1, 0.8]	0.4 [0.1, 1.3]	1.5 [0.3, 6.6]	9.4 [1.6, 35.6]	*
	${px}_{size}>0.5$	0.3 [0.1, 0.9]	0.5 [0.1, 1.3]	2.6 [0.5, 5.3]	5.8 [1.6, 29.9]	*
Clinical	all	0.3 [0.1, 1.0]	0.5 [0.1, 1.5]	2.0 [0.4, 5.8]	7.2 [1.7, 35.5]	*

*The tilt angle is excluded from the clinical validation because this parameter could not be annotated with sufficient accuracy

**Fig. 5:**
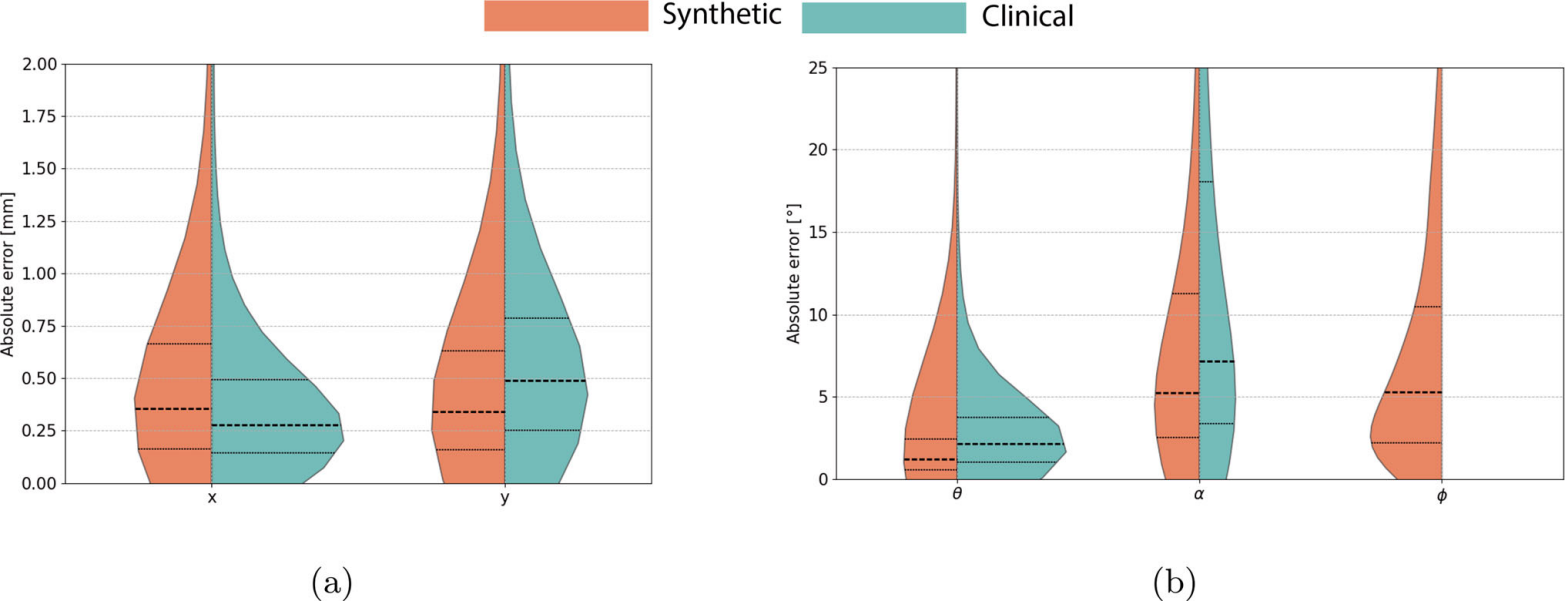
Violin plots showing the absolute error distributions for 5-DoF pose estimation, with dashed lines for the median and dotted lines for the quartile percentiles

Additionally, Table 1 includes the user error relative to the ground-truth values of the synthetic samples. Annotations of the in-plane parameters (*x*, *y*, $\theta$) demonstrate high precision, with a median position error under 0.5mm and a median value of $1.9^{\circ }$ for the plane angle. Out-of-plane annotations ($\alpha$, $\phi$) show a lower accuracy. Although the roll angle has a median error of less than $5^{\circ }$, the $90^{th}$ percentile error reaches $15.0^{\circ }$. The annotation of the tilt angle proves most challenging, with a median error of $12.2^{\circ }$ and a $90^{th}$ percentile extending to $22.3^{\circ }$. Given the high error in the tilt angle annotation, this parameter is excluded from further clinical validation.

### Detection model

The evaluation of the detection model on both synthetic and clinical data is shown in Table 3. The model achieved perfect scores on the synthetic dataset, with a recall of 0.99 and precision and F1 scores that reach 1.00, indicating nearly flawless detection under controlled conditions. For the clinical dataset, the model performance is slightly lower, with a precision of 0.97, a recall of 0.83, and an F1 score of 0.89.

**Fig. 6:**
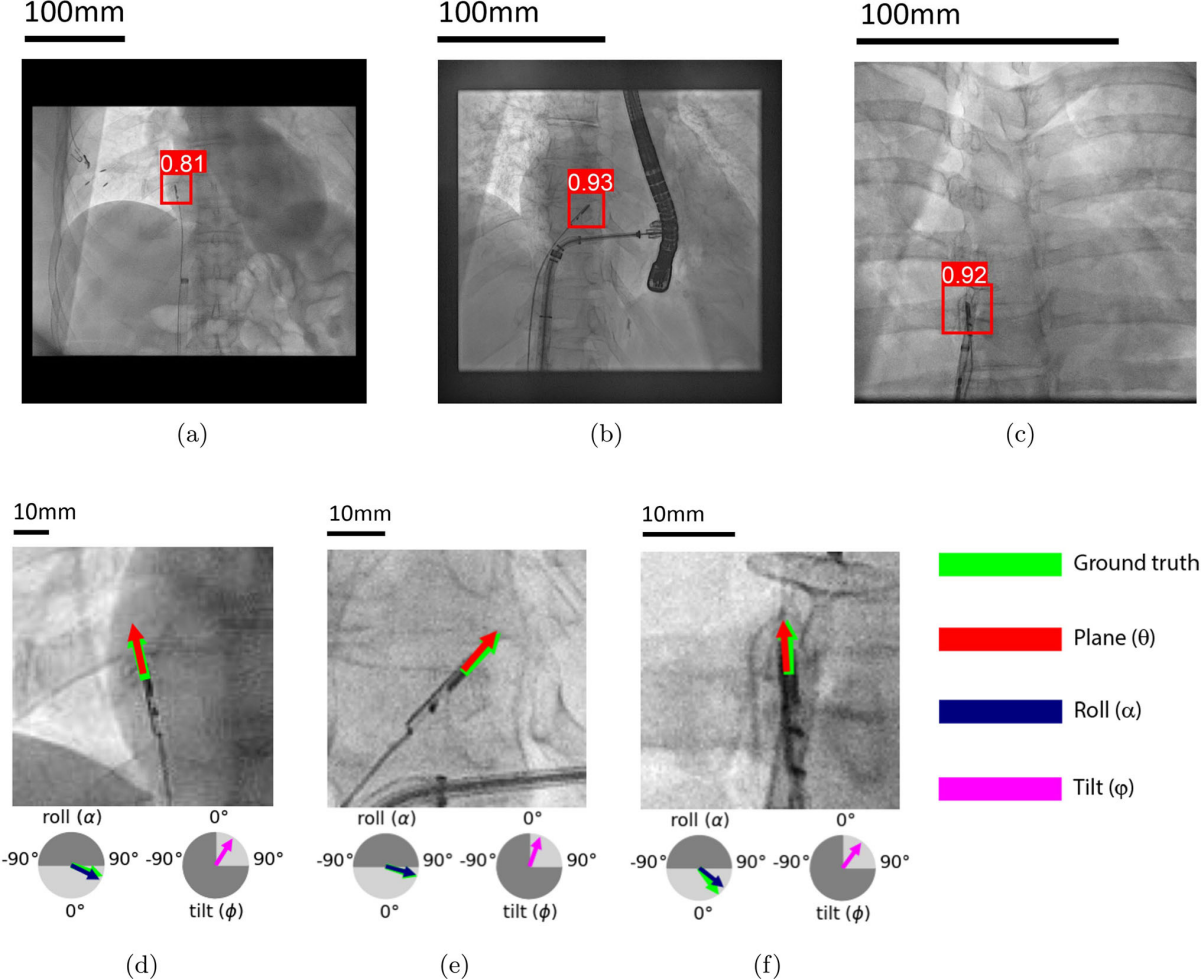
Predictions on test images from the clinical dataset: **(a-c)** detection model with bounding boxes and confidence scores, **(d-f)** pose estimation model with estimated pose parameters, dark gray areas in circles indicate excluded pose estimation ranges. *the tilt angle is excluded from the clinical validation because this parameter could not be annotated with sufficient accuracy

Illustrative examples of three clinical datasets are shown in Fig. 6a–c, with varying pixel sizes, viewpoints, and orientations. In red, the model prediction of the bounding boxes is shown, together with the confidence score.

### Pose estimation model

The absolute errors for all estimated pose parameters can be found in Table 2. Considering the average values across all pixel sizes, the model shows high performance on the synthetic dataset, with a median position error of 0.4mm, a plane error of $1.2^{\circ }$, a roll error of $5.3^{\circ }$, and a tilt error of $5.3^{\circ }$. Compared to the clinical dataset, the model shows similar accuracy in performance for position predictions. However, the median error for the plane angle increased from $1.2^{\circ }$ to $2.0^{\circ }$ and for the roll angle from $5.3^{\circ }$ to $7.2^{\circ }$.

**Table 3:** Performance evaluation of the detection model on synthetic and clinical test data

Test data	Precision	Recall	F1 score
Synthetic	1.00	0.99	1.00
Clinical	0.97	0.83	0.89

The distribution of the pose estimation errors is visualized in violin plots (Fig. 5). In Fig. 5a, violin plots of the absolute error distribution of the positional parameters are shown, while in Fig. 5b, the absolute error distribution of the rotational parameters is shown. The error distribution for the plane and roll angle is more dispersed for the clinical data, as seen in Fig. 5b.

**Fig. 7:**
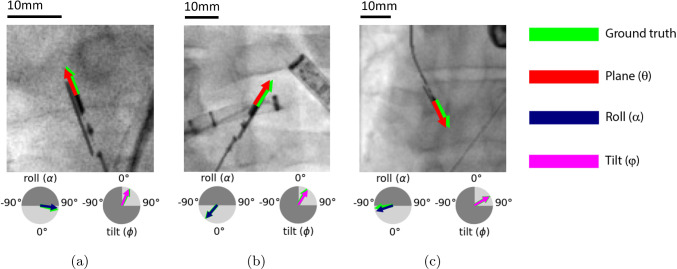
Image patches (100x100 pixels) from the synthetic test set showing pose estimation predictions in different scenarios and pixel sizes: **(a)** no background, **(b)** catheter sheath overlay, **(c)** tilted ICE probe, *dark gray areas in circles indicate excluded pose estimation ranges*

Illustrative examples of pose estimation on synthetic test images can be found in Fig. 7. Predictions on images from the clinical test set can be found in Fig. 6d–f.

## Discussion

In this study, a two-step deep learning-based approach was presented to predict the 5-DoF pose of the ICE probe in real-time. Although ultrasound and XRF fusion has been well established in procedures such as mitral valve repair [18, 19], integrating the ICE probe with XRF images represents a novel contribution.

The use of the ICE probe has gained recognition in recent years as an imaging modality for minimal invasive procedures due to its ability to provide high-resolution imaging of the heart and its surrounding structures while reducing the need for general anesthesia [9, 11]. However, given its limited field of view and the flexibility of the probe, understanding its orientation within the heart simply by observing the acquired images can be challenging. Therefore, for ICE to be applicable and usable as a guiding system for valve repair procedures, image fusion is needed to simplify the interpretation of ultrasound and X-ray imaging. For successful fusion of ICE and XRF, tracking algorithms are required to detect and localize the position and orientation of the ICE probe.

The detection stage was implemented as an initial step, focusing on the ICE probe and thereby eliminating background noise and reducing computational power. The detection model performed well on both synthetic and clinical datasets. It achieved a perfect F1 score of 1.00 on synthetic data and a strong 0.89 on clinical data (see Table 3). The performance gap may be due to reduced variability in synthetic data, which may not capture the complexity of the clinical data. Despite occasional failures in low-contrast images or when the probe is obscured, the model generalizes well, with a precision of 0.97 and recall of 0.83 on the clinical test set. This demonstrates that object detection is an effective first step for the pose estimation model.

The 5-DoF pose estimation model performed well (Table 2), but estimation of the in-plane parameters (position and plane angle) outperformed estimation of the out-of-plane parameters (roll and tilt). This discrepancy stems from using a 2D approach for a 3D problem. Still, the model is capable of estimating out-of-plane rotations with $5.3^{\circ }$ accuracy on synthetic data and $7.2^{\circ }$ on clinical data, across all pixel sizes. Additionally, a performance gap was observed between synthetic and clinical datasets, particularly for the rotation angles. While the prediction of the position parameters was similar across both datasets, with errors in the order of the pixel size, the prediction of the rotation parameters showed more greater differences. This may be due to synthetic training data not fully capturing clinical complexity. This effect may particularly be pronounced for the rotational parameters, which are inherently more challenging to estimate than the positional parameters.

Clinical validation for pose estimation required complex annotations for the out-of-plane parameters, which were therefore less consistent than the in-plane annotations (Table 1). Annotation errors were especially high for tilt angles, leading to the exclusion of this rotation from clinical validation. Despite this, the validation results support the feasibility of pose estimation for the ICE probe and its potential for clinical application.

### Limitations and future outlook

The performance of the framework depends on both pixel size and the presence of overlapping objects. As pixel size increases, the visible size of the ICE probe decreases, resulting in reduced pixel coverage and less information for detection and pose estimation. In Table 2, a trend can be observed that smaller pixel sizes lead to higher model performance for each pose parameter. This is most prominent for the synthetic test set, where significant differences were found between pixel size groups (Appendix 8). In contrast, the clinical test set showed less prominent differences, which suggests that the real-world variability causes more difficulties than a larger pixel size. Additionally, overlapping objects, such as wires or catheter sheaths, further complicate detection and pose estimation. While it would be ideal to detect the probe even when obstructed, such situations are expected to be infrequent or brief in clinical settings.

This paper demonstrates the feasibility of image-based detection and pose estimation of the ICE probe from a single X-ray image. However, the roll and tilt angles cannot be fully estimated from 2D X-ray images due to the symmetry of the probe (Fig. 4). Future work will focus on addressing the symmetry issue. One potential solution is the use of biplane XRF imaging, as discussed in Annabestani et al. [21]. However, biplane imaging increases radiation exposure for patients and is not a standard imaging technique for valve therapy procedures. An alternative approach is adding fiducial markers to the ICE probe, which in turn comes with modifications of the hardware.

**Fig. 8:**
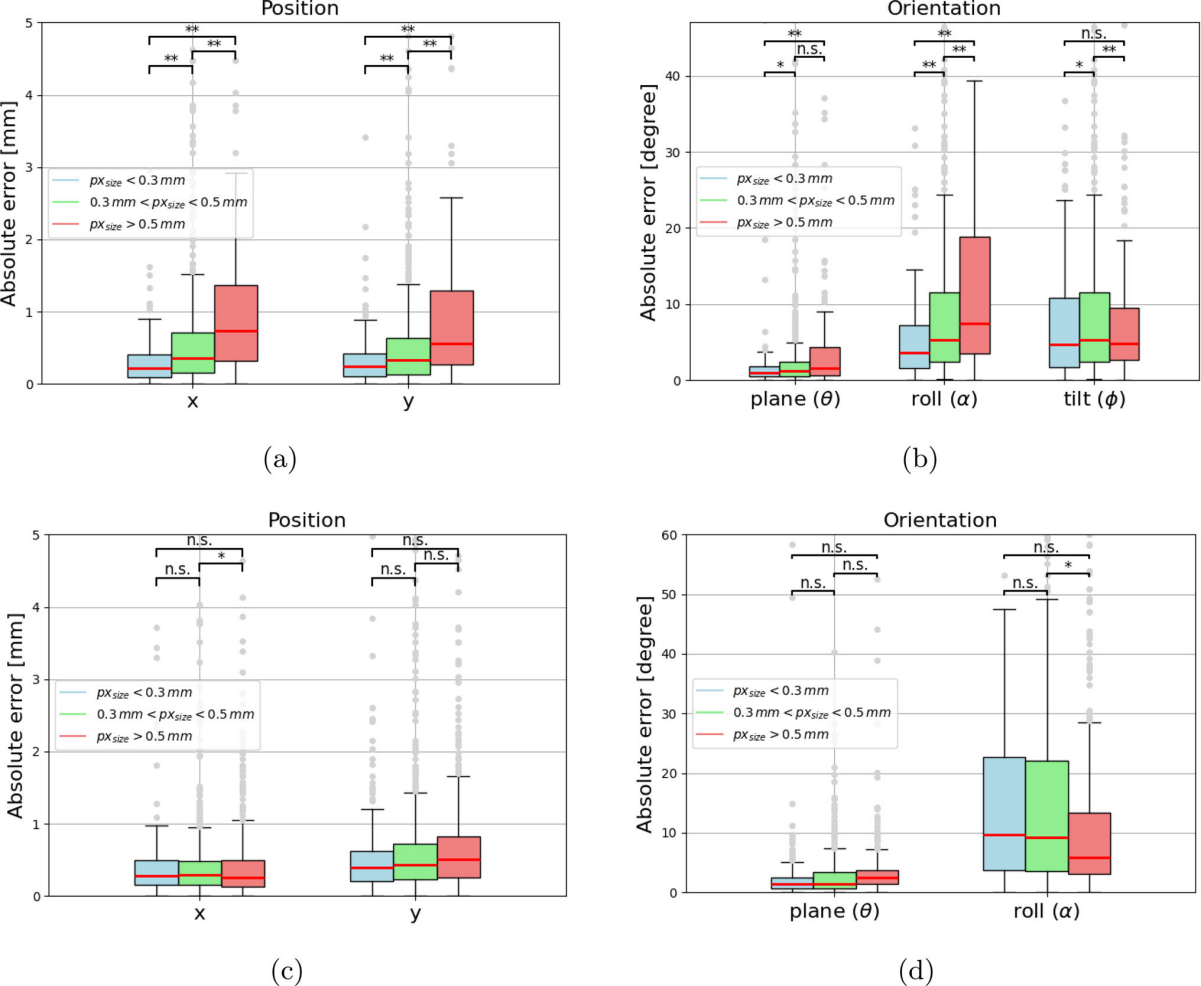
Performance evaluation for each degree of freedom grouped per pixel size for **(a,b)** the synthetic test set and **(c,d)** the clinical test set, with n.s. no significant statistical differences, * $p_{value}$<0.05, and ** $p_{value}$<0.01

This study focused on estimating 5-DoF for the ICE probe, excluding the third positional parameter, depth (*z*), which cannot be directly obtained from 2D X-ray images. For a depth translation of 1 cm, the visible difference in the size of the ICE probe is at most half a pixel ($\sim$0.1mm). This holds for ideal circumstances where the tilt angle is equal to zero. Increased tilt angles result in probe foreshortening, complicating depth estimation even further. Future research will explore alternative approaches, such as incorporating table height, C-arm angle, or multiple projections, to estimate depth.

The proposed tracking system is a promising step toward integrating ICE with real-time navigation, with detection and pose estimation models achieving inference times of around 10 ms using NVIDIA T1200, enabling real-time tracking. We envision this framework enabling real-time fusion of ICE with live X-ray images, similar to the EchoNavigator product (Philips Healthcare, Best, The Netherlands), which provides real-time echo and X-ray fusion for specific TEE probes.

### Conclusions

To conclude, a two-step deep learning framework is presented to detect the ICE probe and estimate its 5-DoF pose in 2D XRF images. Synthetic and clinical validation showed the feasibility of real-time image-based tracking of the ICE probe. This approach facilitates the use of ICE in valve therapy, which may improve procedural accuracy and broaden the use of minimally invasive treatments for valve repair procedures.

## Data Availability

The authors do not have permission to share data.

## Appendix A

Performance evaluation by grouped pixel size (Fig. 8), with indicated significant differences (Table 4).

**Table 4:** P-values to determine statistical significance of the absolute error between the groups divided by pixel size, as described in Sect. 2.8, with group 1: $px_{size}$<0.3mm, group 2: 0.3mm<$px_{size}$<0.5mm, group 3: $px_{size}$>0.5mm (n.s not significant)

Test data	Compared groups	x	y	$\theta$	$\alpha$	$\phi$
	1 and 2	$<0.01$	$<0.01$	$<0.05$	$<0.01$	$<0.05$
Synthetic	1 and 3	$<0.01$	$<0.01$	$<0.01$	$<0.01$	n.s
	2 and 3	$<0.01$	$<0.01$	n.s	$<0.01$	$<0.01$
	1 and 2	n.s	n.s	n.s	n.s	*
Clinical	1 and 3	n.s	n.s	n.s	n.s	*
	2 and 3	$<0.05$	n.s	n.s	$<0.05$	*

*The tilt angle is excluded from the clinical validation because this parameter could not be annotated with sufficient accuracy
